# Reply: Early onset of breast cancer in British black women

**DOI:** 10.1038/sj.bjc.6604416

**Published:** 2008-06-10

**Authors:** R L Bowen, S W Duffy, D A Ryan, I R Hart, J L Jones

**Affiliations:** 1 Institute of Cancer And Cancer Research UK Clinical Centre, Centre for Tumour Biology Barts and the London Queen Mary's School of Medicine and Dentistry John Vane Science Centre, Charterhouse Square London, London, EC1M 6BQ UK

**Sir**,

We thank the authors for their interest in our study and apologise to them for having omitted citing their study (Wild *et al*, 2006) in our original paper. We did, however, make reference to their paper in our recent letter to the *British Journal of Cancer* (Bowen *et al*, 2008, in press). We note that this study found increased breast cancer mortality in those women born in West Africa and domiciled in the United Kingdom (not necessarily black African) whereas we found increased mortality in British black women but only in those with small tumours. In this recent letter, we have also described more fully the ethnicity of the black women in our cohort and while almost half were of Caribbean descent, at least one-third were black African or British.

It is not clear that an analysis in the United Kingdom based on country of birth is comparable to one, such as ours, based on self-reported ethnicity. Also, by limiting our study to one area of East London and one referral hospital we have been able to control for socioeconomic status and variations in treatment practise and, thus, the outcome.

We would also like to defend our age-related observations, even with the broad age groupings available to us. It was clear that the disease indeed does occur at younger ages, accounting for the different age ranges of the population, in the black population in the area of our study. We consider, therefore, that our conclusion is not a mistake although we do accept that our findings need to be substantiated in a national study of breast cancer using self-reported ethnicity to characterise the patterns of breast cancer better in British black women.
